# Research Note: Relationships between texture and water property measurements in raw intact broiler breast fillets with the wooden breast condition

**DOI:** 10.1016/j.psj.2024.103830

**Published:** 2024-05-08

**Authors:** Bin Pang, Jian Zhang, Brian Bowker, Yi Yang, Jingxin Sun, Xiao Sun, Jianteng Wei, Hong Zhuang

**Affiliations:** ⁎ College of Food Science and Engineering, Qingdao Agricultural University, Qingdao 266109, China; † USDA, Agricultural Research Service, U.S. National Poultry Research Center, Athens, GA 30605; ‡ Institute of Animal Husbandry and Veterinary Medicine, Beijing Academy of Agriculture and Forestry Sciences, Beijing 100097, China; § School of Computer and Artificial Intelligence, Beijing Technology and Business University, Beijing 100048, China; # School of Biological Science and Food Engineering, Chuzhou University, Chuzhou 239000, China

**Keywords:** MORS, myofibrillar water, NMR, T_2_ relaxation, woody breast

## Abstract

Relationships between texture measurements and meat water properties were investigated in raw intact broiler breast fillets with the wooden breast (**WB**) condition. Texture measurements included subjective WB scores and blunt Meullenet-Owens Razor Shear (**BMORS**). Water properties were determined with low-field nuclear magnetic resonance (**LF-NMR**). Spearman correlation was used to estimate relationships between WB scores and water properties, while Pearson correlation was used for relationships between BMORS force and water properties. LF-NMR measurements exhibited 3 water components: protein-associated or hydration water T_2b_, intra-myofibrillar water or immobilized water T_21,_ and extra-myofibrillar water or free water T_22_ in chicken breast meat. Significant and strong Spearman correlations were found between the WB scores and T_21_ time constant, the abundance (normalized areas) of T_22_, and the proportion of T_21_ and T_22_ (*r_s_* > 0.60, *P* < 0.001). Strong Pearson correlations (*r* = 0.72) were noted only between the T_21_ time constant and BMORS force. These results demonstrate that water may contribute to the specific texture characteristics measured with subjective WB scoring (palpable hardness and rigidity) and BMORS (hardness and share force) in raw broiler breast fillets with the WB condition.

## INTRODUCTION

The wooden breast (**WB**) condition is an emerging muscle abnormality, which significantly alters physical as well as biochemical properties in raw broiler breast fillets (pectoralis major). The distinct difference between the WB fillets and normal ones is tactile characteristics in raw meat with the WB fillets being much harder and more rigid (Pang et al., 2020). Several methods have been used to measure or characterize the unique texture properties of raw WB muscle, including subjective scoring, compression force, texture profile analysis (**TPA**), and Meullenet-Owens Razor Shear (**MORS**). Among them, subjective scoring is the predominant method. This method defines WB meat and/or its severity based on tactile properties of palpable hardness and rigidity of raw breast meat (Pang et al., 2020). The MORS method also successfully separated raw normal samples from severe WB samples (Pang et al., 2020). The texture properties of meat measured with MORS methods depend upon the type of razor blade used. A sharp blade (a regular MORS method) mainly measures shear force of muscle; however, a blunt blade, measures the combination of shear and compression resistance of raw meat muscles (Pang et al., 2020). Therefore, the data collected with MORS methods indicate that the WB condition also alters the shear resistance of broiler breast meat in addition to hardness. Changes in connective tissue or collagen content, composition, and structure in muscle tissue have been believed to be responsible for the tactile characteristics of WB meat [Velleman, 2020]. However, evidence does not always support the hypothesis. There was the lack of relationships between meat stiffness and contents of total collagen, insoluble, and intramuscular connective tissue in the WB meat in the early postmortem period (Zhu et al., 2023). Hydroxylysylpyridinoline concentration, the principle non-reducible crosslink in fibrilla collagen and a critical factor in tissue stiffening, was not affected by the WB condition in the Ross 308 strain (Velleman, 2020; Baldi et al., 2019). In addition, research also indicates a relationship between water and severity of the WB condition in raw fillets. With a subsample, Tasoniero et al. (2017) showed that there were positive correlations between texture characteristics (represented by compression force) and meat water properties in raw broiler breast meat with the WB condition. The losses in the WB hardness during storage have been ascribed to water losses in meat by Sun et al. (2018).

Proton nuclear magnetic resonance (**NMR**) relaxation times have been widely used to investigate water properties (including water distribution or population, mobility, and abundance) in a variety of materials and the relationship between water properties and texture properties in meat. Bertram et al. (2005) found that overall, the NMR relaxation times explained a high percentage of variation in measured sensory texture attributes of cooked pork samples (76% for meat tenderness, 77% for chewing time, and > 70% for juiciness). Research has consistently demonstrated much greater water content in the WB fillets compared with the normal ones (Soglia et al., 2016). With low-field ^1^H NMR (LF-NMR), Soglia et al. (2016) demonstrated the WB condition not only resulted in increased palpable hardness but also altered NMR relaxation times or water properties/distribution in raw WB meat. Therefore, the objective of this study was to investigate the relationship between water properties, measured with LF-NMR, and texture characteristics, measured with subjective WB score and BMORS, in raw intact broiler breast fillets with the WB condition.

## MATERIALS AND METHODS

### Sample Preparation and Texture Measurements

Fillet samples as well as the WB sores and BMORS measurements, which were used for this study, were collected by Zhang et al. (2021) in a trial for comparison of different shear methods for WB texture measurements. They were briefly summarized as below. A total of 72 boneless skinless breast fillets (pectoralis major) of broilers (8–9 wk old) were collected from the deboning line of a commercial processing plant (Wayne Farms LLC, Pendergrass, GA) at approximately 3 h postmortem. Fillets were scored with a 3-point scale (1 = normal; 2 = moderate WB; 3 = severe WB) based on palpable hardness and rigidity (Pang et al., 2020) before they were individually vacuum-packed and stored overnight at 4℃ (24 for each group). The Blunt Meullenet-Owens Razor Shear (**BMORS**) peak force was measured using a Texture Analyzer (model TA-XT-Plus, Texture Technologies Corp., Hamilton, MA) with a 490N loading cell (Zhang et al., 2021). For each fillet, 5 BMORS measurements were conducted. The average value of the 5 measurements was used for correlation analysis.

### Low-Field NMR Measurements

A ^1^H-NMR analyzer (LF 90II Proton-NMR, Bruker optics, Woodlands, TX) was used to measure transverse relaxation (T_2_) of raw fillets by the Carr-Purcell-Meiboom-Gill (**CPMG**) pulse sequence with the settings: τ-value (90–180° pulse separation) = 1 ms, 200 echoes, and 16 scans. Fillet temperature at measurements was approximately 4℃. The CPMG decay curves were processed with the CONTIN regularization algorithm provided by Minispec (contin-ilt_mq_nf, Bruker Biospin GmbH, Rheinstetten, Germany), resulting in the corresponding water properties, time constants (T), their relative areas (P), and their normalized areas (A) of hydration water (T_2b_, P_2b_, and A_2b_), intra-myofibrillar water (T_21_, P_21_, and A_21_), and extra-myofibrillar water (T_22_, P_22_, and A_22_).

### Statistical Analysis

Descriptive statistics of raw data were conducted with SAS Proc means for WB scores, BMORS peak force, and water property parameters T, A, and P. The relationships between WB scores and meat water properties (T_2b_, T_21_, T_22_, A_2b_, A_21_, A_22_, P_2b_, P_21_, and P_22_) were analyzed by calculating Spearman correlation coefficients (*r_s_*) and the relationships between BMORS force and water properties by calculating Pearson correlation coefficients (*r*) using SPSS software (version 25.0). For discussion, the following descriptors were used to determine the relative strength of the correlations: weak (|*r|* = 0.20-0.39), moderate (|*r|* = 0.40-0.59), strong (|*r|* = 0.60-0.79), and very strong (|*r|* = 0.80-0.99).

## RESULTS AND DISCUSSION

Descriptive statistics showed that the coefficients of variation [CV, equal to 100 x (Standard Deviation/Mean)] were 41% for WB scores and 57% for BMORS force measurements (Table 1). They ranged from 10% (T_2b_ and T_21_) to 57% (P_2b_ and A_22_), varying with water property parameter. The CV permits the comparison of variates free from scale effects. Therefore, the CV results indicate that the variations in measurements of textures and water characteristics used in this study.

**Table 1:** Descriptive statistics of **WB** scores, BMORS measurements, and water properties of raw broiler breast fillets.

Parameter	N	Min	Max	Mean	SD	CV (%)
WB score	72	1	3	2	0.82	41
BMORS force (N)	72	9.73	84.38	30.77	17.56	57
T_2b_ (ms)^1^	72	0.34	0.46	0.42	0.04	10
T_21_ (ms)^1^	72	44.2	67.2	53.8	5.5	10
T_22_ (ms)^1^	72	156.2	301.9	217.8	24.0	11
A_2b_ (area/100g)^1^	72	1.30	5.21	2.71	1.01	37
A_21_ (area/100g)^1^	72	225.9	519.1	393.3	46.2	12
A_22_ (area/100g)^1^	72	46.3	510.1	199.3	114.2	57
P_2b_ (%)^1^	72	0.20	1.43	0.51	0.29	57
P_21_ (%)^1^	72	45.27	86.85	68.24	11.56	17
P_22_ (%)^1^	72	12.05	54.45	31.24	11.78	38

WB, wooden breast; BMORS, blunt Meullenet-Owens Razor Shear; SD, standard deviation; and CV, coefficient of variation, equal to 100 x SD/Mean.

1 T represents time constant (ms); P represents proportion (%); A represents normalized area (area/100g fresh weight of meat); 2b in subscript represents bound water or hydration water with constant time 4.3–5.1 ms; 21 represents intra-myofibrillar water or immobilized water with constant time 35–51 ms; and 22 represents extra-myofibrillar water or free water with constant time >82 ms.

The relationships between texture measurements and water property parameters were analyzed with Spearman and Pearson correlation coefficients (Table 2). Significant Spearman and Pearson correlation coefficients (|*r*_s_| = 0.45-0.78, *P* < 0.001) were observed between subjective WB scores and meat water properties and between BMORS force and water properties, respectively, except for A_21_. The strong Spearman correlations were found for A_22_, P_21_, P_22_, and T_21_ (r = 0.74, -0.78, 0.77, and 0.69, respectively) and strong Pearson correlations (*r* = 0.72) for T_21_. These results indicate a strong association between changes in extra-myofibrillar water abundance (A_22_ and P_22_), intra-myofibrillar water mobility (T_21_), and relative water abundances (P_21_) and subjective WB scores (or the palpable hardness and rigidity of broiler breast meat) and between changes in intra-myofibrillar water mobility (T_21_) and BMORS texture measurements in broiler breast meat with the WB condition. Additionally, the *r* values for T_2_ parameters T_2b_, T_21_, T_22_, A_2b_, A_21_, and P_2b_ in Pearson analyses were similar to the *r_s_* values between WB score and those T_2_ parameters (< 0.11); however, for T_2_ parameters A_22_, P_22_, and P_21_, the *r* values with BMORS were much smaller than those with WB scores (> |0.25|). These results suggest that extra-myofibrillar water may be responsible for the specific texture properties, such as rigidity, assessed by the subjective WB scoring method.

**Table 2:** Correlation coefficients between subjective WB score/BMORS force and water properties in raw broiler breast fillets.

	Time constant (ms)			Normalized area (area/100g)			Relative area (%)		
	T_2b_	T_21_	T_22_	A_2b_	A_21_	A_22_	P_2b_	P_21_	P_22_
WB score^1^	0.46^⁎⁎⁎^	0.69^⁎⁎⁎^	0.54^⁎⁎⁎^	−0.55^⁎⁎⁎^	0.21	0.74^⁎⁎⁎^	−0.60^⁎⁎⁎^	−0.78^⁎⁎⁎^	0.77^⁎⁎⁎^
BMORS Force	0.45^⁎⁎⁎^	0.72^⁎⁎⁎^	0.46^⁎⁎⁎^	−0.47^⁎⁎⁎^	0.27	0.49^⁎⁎⁎^	−0.49^⁎⁎⁎^	−0.50^⁎⁎⁎^	0.50^⁎⁎⁎^

* *P* ≤ 0.05, ^⁎⁎^*P* ≤ 0.01, ^⁎⁎⁎^*P* ≤ 0.001.

1 WB score 1 = normal, 2 = moderate WB, and 3 = severe WB. WB, wooden breast; BMORS, blunt Meullenet-Owens Razor Shear.

Texture is a critical quality attribute for meat and has been studied intensively in past. Three major factors that impact texture toughness are sarcomere length, connective tissue content, and the extent of postmortem proteolysis. Since the texture hardness of broiler breast with the WB condition occurs in live birds, sarcomere length and connective tissue have been investigated for elucidating the cause of WB texture characteristics. In poultry, toughness of breast fillets deboned before rigor mortis is attributed to shortening of sarcomeres. Several studies were conducted to investigate if shortening of sarcomere length resulted in hardness of WB-affected muscles. Results demonstrate consistent longer sarcomere length in WB fillets than that in normal fillets and suggest that sarcomere shortening postmortem is not responsible for hardness observed in WB (Soglia et al., 2020). On the other hand, investigations of connective tissues in broiler fillets show marked differences in collagen composition, structure, and content between normal and WB fillets and lead to the hypothesis that the fibrotic deposition and organization of extracellular matrix proteins, especially the fibrillar collagens, may be responsible for the phenotype of the WB fillets (Velleman, 2020). However, evidence indicate that other factors may also contribute to tactile properties of WB meat. Overall, compression force measurements were better correlated with WB severity than shear force (Pang et al., 2020). During postmortem refrigerated storage, WB fillets lose hardness significantly (Sun et al., 2018; Zhu et al., 2023). However, amount of intramuscular connective tissue in severe WB were hardly changed (Hasegawa et al., 2020; Zhu et al., 2023). Moisture losses was associated with reduced hardness of WB fillets during refrigerated storage (Sun et al., 2018).

Pearce et al. (2011) concluded that, in meat, an increase in the P_21_ indicated an increase in the number of protons (essentially water) within the myofibrils in the intra-myofibrillar space; an increased P_22_ indicated a higher number of protons and therefore an increase in the extra-myofibrillar water population; an increase in T_21_ represented an increased relaxation time of water in the intra-myofibrillar space, which might be due to an increase in the spacing between the myofibrils; and an increase in the T_22_ time constant represented an increase in the space in the extra-myofibrillar area. Therefore, correlation analyses from the present study indicate that both water abundance (indicated by both proportion and normalized area) and spaces in muscle fibers may be involved in the tactile characteristics of the WB fillets. Increased water proportion within the intra-myofibrillar space negatively but increased water proportion within the extra-myofibrillar space positively affects tactile characteristics of WB meat. The abundance of the intra-myofibrillar water (A_21_) does not appear to influence the tactile properties of the raw WB fillets; however, texture characteristics, especially subjective hardness and rigidity of WB fillets, may be more likely impacted by the abundance of the extra-myofibrillar water (A_22_). In addition, the space in the intra-myofibrillar compartment was strongly and positively related to texture measurements of WB muscle regardless of the methodology. In the other words, our Pearson correlation analyses indicate that myofibrillar structure may be responsible for the common texture properties measured with both subject WB scoring and shear force; however, water in meat may be involved in the texture properties that are hardly captured by a shear method, such as rigidity in chicken fillets with the WB condition. It has been well-demonstrated that changes in the sarcomeres results in rigor mortis or stiffening of muscle and also changes texture of meat postmortem. Therefore, it is not surprising that the alteration in myofiber structure is involved in changes in texture of broiler breast meat with the WB condition.

It has been noted that there are relationships between changes in water properties measured with NMR T_2_ relaxation methods and texture characteristics in meat. With lamb *M. Longissimus dorsi*, Pearce et al. (2008) investigated relationships between an instrumental texture measurement (shear force) and water properties measured with an NMR instrument. They also found that the decrease in the shear force was accompanied by decreases in T_21_ and P_22_ and increases in P_21_ regardless of treatment during postmortem aging. The chemometrics modeling revealed a relationship of r = 0.78 between shear force and NMR relaxation measurements. It was concluded that a high amount of intra-myofibrillar water and a low amount of extra-myofibrillar water may be associated with more tender meat. With pork *M. Longissimus dorsi*, Bertram et al. (2005) investigated relationships between sensory texture attributes and the intrinsic water properties in the meat using continuous LF-NMR proton T_2_ relaxometry during cooking of meat. Multivariate sensory variable analysis revealed that the distributed NMR T_2_ relaxation times explained a high percentage of the variation in all the measured sensory attributes, juiciness, hardness, tenderness, crumbliness, and chewing time. These published data demonstrated that there were relationships not only between T_2_ measurements and instrumental texture properties but also between T_2_ measurements and sensory texture attributes in meat. In agreement with these findings, the present study further demonstrates that there are relationships between water properties and the changes in texture characteristics within raw broiler breast meat with the WB condition and relations vary with T_2_ measurements.

Water could play a very important role in the mechanical properties of living organisms. In plants, the firmness of fruits and leaves is well associated with the tissue water status and has been attributed to turgor pressure, or pressure generated when cell wall is pushed by inside water against the cell wall, which causes rigidity of the cell. Here we hypothesize that this could be a case for the cells within wooden breast meat, although turgor pressure is generally believed not to occur in normal animal cells (because without a cell wall, too much water inside animal cells would cause normal animal cells to lyse). In addition to consistently greater connective tissues (Velleman, 2020; Zhu et al., 2023), the WB condition also affects collagen fibril structure with parallel packing of collagen monomers, smaller average collagen fibril diameter and longer average collagen D-period, and high levels of collagen crosslinking, which results in tightly packed collagen fibrils and reduces the flexibility (Velleman, 2020). These changes may also prevent muscle cells from lysing when water accumulates inside cells and also inside muscle fiber bundle to some degree and further result in increased hardness and rigidity of broiler breast fillets. This is consistent with data in published reports that the WB meat contains more moisture and has greater abundance of water in the extra-myofibrillar compartment (Soglia et al., 2016; Tasoniero et al., 2017) and changes in hardness were associated with water loss during storage (Sun et al., 2018; Zhu et al., 2023). It is also supported by our findings in this study that increasing severity of the WB condition was positively associated with increasing space in both the intra-myofibrillar and extra-myofibrillar compartments and abundance of the extra-myofibrillar water. The further demonstration is warranted to investigate the effect of water on mechanical properties, such as hardness and stiffness of animal cells with changes in collagen content, composition, and structures. The investigation of the changes in connective tissue (including proteoglycans, collagens, and non-collagenous glycoproteins) during early postmortem storage could also provide the key information about our hypothesis.

In conclusion, while the differences in histology and collagen content have been hypothesized to be responsible for specific texture properties of raw WB meat, the data from the present study suggest that water might also contribute to the unique texture characteristics of the WB meat or the severity of the WB condition in raw broiler fillets. Further studies are needed to demonstrate the relationship between changes in water properties and connective structures as well as compositions and the effect of interaction between water properties and connective tissues on raw meat hardness and rigidity.

## DISCLOSURES

The authors declare no conflicts of interest.
